# MoBILAB: an open source toolbox for analysis and visualization of mobile brain/body imaging data

**DOI:** 10.3389/fnhum.2014.00121

**Published:** 2014-03-05

**Authors:** Alejandro Ojeda, Nima Bigdely-Shamlo, Scott Makeig

**Affiliations:** Swartz Center for Computational Neuroscience, Institute for Neural Computation, University of California San DiegoLa Jolla, CA, USA

**Keywords:** EEG, motion capture, mobile brain/body imaging, MoBI, EEGLAB, multimodal neuroimaging

## Abstract

A new paradigm for human brain imaging, *mobile brain/body imaging* (MoBI), involves synchronous collection of human brain activity (via electroencephalography, EEG) and behavior (via body motion capture, eye tracking, etc.), plus environmental events (scene and event recording) to study joint brain/body dynamics supporting natural human cognition supporting performance of naturally motivated human actions and interactions in 3-D environments (Makeig et al., 2009). Processing complex, concurrent, multi-modal, multi-rate data streams requires a signal-processing environment quite different from one designed to process single-modality time series data. Here we describe MoBILAB (more details available at sccn.ucsd.edu/wiki/MoBILAB), an open source, cross platform toolbox running on MATLAB (The Mathworks, Inc.) that supports analysis and visualization of any mixture of synchronously recorded brain, behavioral, and environmental time series plus time-marked event stream data. MoBILAB can serve as a pre-processing environment for adding behavioral and other event markers to EEG data for further processing, and/or as a development platform for expanded analysis of simultaneously recorded data streams.

## Introduction

For nearly 50 years the dominant approach to cognitive EEG experiment protocols and subsequent data analyses has been the Event Related Potential (ERP) paradigm in which EEG epochs are extracted from the continuous EEG data time-locked to one or more classes of experimental events (typically, stimulus onsets or finger button presses). Event-locked averages of these epochs (ERPs) extract the relatively small portion of the EEG that is both time-locked and phase-locked to the events of interest (Makeig et al., 2004). The same paradigm can be extended to linear transforms of the channel data including its maximally independent component processes (Makeig et al., 1996, 2002), and/or to time/frequency transforms of these EEG time series (Makeig, 1993; Tallon-Baudry et al., 1996; Delorme and Makeig, 2004).

The ERP paradigm assumes that differences in EEG dynamics across event-related trials unrelated to the experimental events of interest can be eliminated through random phase cancellation by averaging a sufficient number of event time-locked epochs. To maximize the effectiveness of this assumption, participants in ERP experiments are typically instructed to sit still and to minimize blinks and other muscle activities while performing some task involving evaluation of presented stimuli, the participant indicating his or her stimulus decisions by pressing a finger button (or “microswitch”). During data analysis these button press responses are considered to be in effect (point) processes without spatial or temporal extent. However, the instruction to refrain from blinking and making any other extraneous movement is in effect a dual-task that forces the brain to operate under unnatural and somewhat stressful circumstances (Verleger, 1991). It also severely restricts the range of task paradigms and behaviors that can be employed to observe and understand how human brain dynamics support our natural behavior and experience.

A new direction in experimental paradigm design was proposed by Makeig et al. (2009) to enable, for the first time, measurement and analysis of human brain dynamics under naturalistic conditions including subject eye and motor behavior in 3-D environments. Compared to previous modes of functional brain imaging, the new concept in effect proposed a new brain imaging modality that Makeig et al. termed *mobile brain/body imaging (MoBI)*. In this paradigm, synchronized streams of behavioral and environmental time series data are measured along with subject EEG and/or other brain and physiological signals. In many practical circumstances, data collection rates may differ and some information streams may be sampled irregularly. Combining data modalities as different as motion capture, eye tracking, sound and video recordings, etc., with high-density EEG data allows study of brain dynamics under conditions much closer to everyday life. To date the MoBI paradigm has been applied in studies of brain dynamics supporting gait, balance, and cognition during walking (reviewed in Gramann et al., 2011; Sipp et al., 2013) and to study expressive gesturing (Leslie et al., in press).

Traditional scalp-channel ERP analysis can be carried out in almost all available EEG toolboxes including EEGLAB (Delorme and Makeig, 2004), Brainstorm (Tadel et al., 2011), FieldTrip (Oostenveld et al., 2011), BrainVision Analyzer, and SPM (Friston et al., 1994). However, since EEG software has most often been designed to handle unimodal EEG data (plus one or more event-marker channels), a new tool set is needed to deal with the complex analysis problems involved in efficient analysis of multi-modal MoBI data.

A related experimental EEG paradigm of increasing research interest is the development of brain-computer interface (BCI) models and applications (Makeig et al., 2012). In this paradigm, brain activity associated with a cognitive state or task is used to estimate a model of a subject's cognitive brain state, response, or intent with a goal of classifying or estimated current or future responses so as to control external interfaces (Schneider and Wolpaw, 2012). When the subject (or an external interface) requires a timely interpretation of current brain/behavior state or intent, a near real-time pipeline is needed to process the data and estimate and/or update the model continuously. Near real-time signal processing may impose serious computational constraints on the signal processing methods that can be used, limiting the type and range of states estimated and the number of data streams processed (Wang and Jung, 2013). Basic studies of brain dynamics, on the other hand, may use latent variables in complex BCI models trained off-line to model brain state or dynamics associated with subject brain states, responses, or intent during an EEG or MoBI experiment. Typically, dynamic features used in such models are optimally selected in some fashion from a large repertoire of possible features. Examination of the model features so selected may be termed informative feature analysis. Current leading edge BCI toolkits include BCILAB (Kothe and Makeig, 2013) and BCI2000 (Schalk et al., 2004). Other EEG toolboxes such as MNE (Gramfort et al., 2014) and FieldTrip provide basic support for real-time signal processing as well. BCILAB, in particular, provides a strong basis for informative feature analysis of recorded EEG and/or multi-modal data.

The MoBILAB toolbox introduced here is designed to support analysis and visualization of any number of concurrently recorded multimodal data streams during off-line analysis. To extend human EEG analysis tools to encompass new modes of analysis of multiple physiological and behavioral data modalities while making the basic software functions easy to use and build upon, MoBILAB exploits the most recent advances in object-oriented programming supported by MATLAB Versions 7.5 and above. To learn more about MATLAB object oriented programming, see MathWorks on-line documentation under mathworks.com/help/matlab at matlab_oop/classes-in-the-matlab-language.html.

## Software architecture

**Figure 1:**
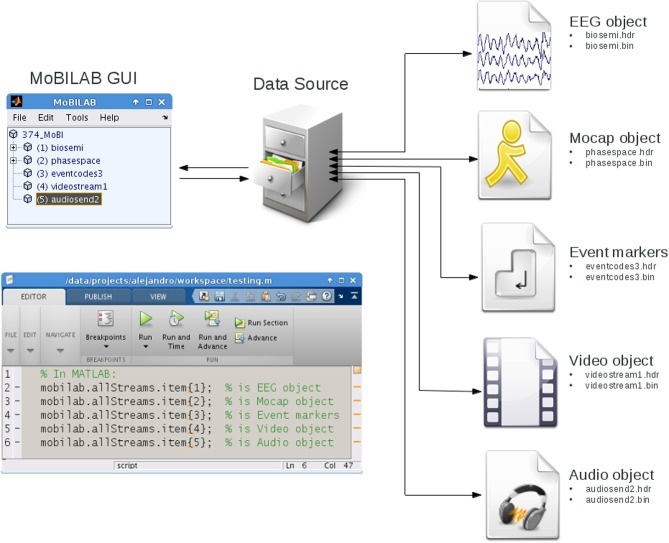
**The MoBILAB toolbox architecture.** From left to right: through the MoBILAB GUI (upper left), the user can read new or previously processed data sets (right) in one of several formats [currently Lab Streaming Layer (.xdf), EEGLAB (.set), or MoBILAB (.hdr,.bin)] and instantiates a corresponding *dataSource* object that identifies the constituent data streams and creates stream-specific objects to handle them. Each data stream object defines methods for processing and visualization of the type of data it contains. The connecting arrows indicate that each MoBILAB module can communicate bi-directionally with any other object it interfaces. Each data stream object handles two files, a header that provides metadata about its properties, and a binary data file that is memory mapped to disk, allowing its data (no matter how large) to be accessed through its *data* field.

MoBILAB is composed of three independent functional modules: (1) the Graphic User Interface (GUI), (2) a data objects container, and (3) any number of modality-specific data stream objects (or stream objects). These three modules decouple GUI, file I/O, and data processing, thus allowing extensions and/or re-implementation of parts of the toolbox without making dramatic changes to the whole system, e.g., preserving large, still-stable parts of the code. Figure 1 below shows a schematic of the MoBILAB architecture.

The MoBILAB GUI is controlled by a “*mobilabApplication*” object called “*mobilab*,” this object creates the interactive tree in which the raw data stream objects and its descendants are represented; it also assigns a modality-specific menu item to each object.

The data object container is implemented in the class “*dataSource*”; this object imports a multimodal data file and collects modality-specific data stream objects in a cell array that is stored in the object property *item*. As a container, the *dataSource* object defines easily applied methods for deleting and inserting stream objects that take care of updating the logical pointers encoding parent-child relationships among the elements of the tree. To read a new file format, for example, a new class can be derived inheriting from *dataSource* its basic functionalities while implementing only the format-specific data reading method.

Natively, MoBILAB reads multimodal data files in the *extensible data format* (XDF) (freely available at code.google.com/p/XDF) designed to work both with MoBILAB and with the Lab Streaming Layer (LSL) data acquisition and control framework of Christian Kothe (Delorme et al., 2011), software freely available at code.google.com/p/labstreaminglayer. To import.*xdf* files produced by LSL during MoBI experiments, MoBILAB defines the child class “*dataSourceXDF*.” When an.*xdf* file is imported by *dataSourceXDF*, a set of header and binary files are created for each data stream read from the source.*xdf* file. Data stream objects are then constructed that map the metadata and data samples contained in the header and binary data files, respectively, onto object properties. Data sample mapping uses memory-mapping (detailed below). This mapping between object properties and files allows automatic file updating by modifying stream object properties. The *dataSource* object is stored in *mobilab* property *allStreams* and can therefore be accessed from the MATLAB workspace as *mobilab.allStreams*.

**Figure 2:**
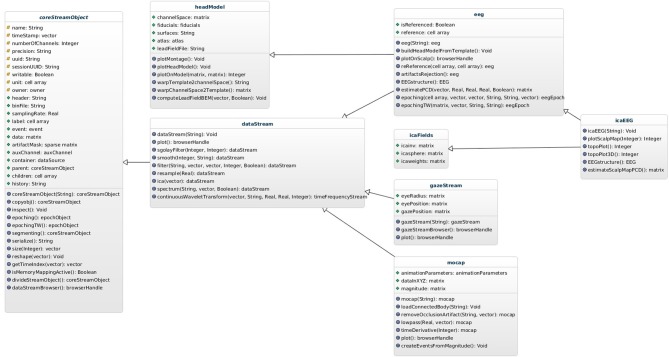
**The *dataStream* class hierarchy.** From left to right: the base class *coreStreamObject*. This is the common ancestor of all data stream objects, it defines the underlying machinery for storing data in a memory-mapped source file and accessing it as a matrix though its *data* property. Next (right) in the hierarchy is the *dataStream* class that defines methods for processing generic time series data including data resampling, digital filtering, independent component analysis, etc.. From this are derived specific data stream classes including *eeg* for EEG data, *mocap* for motion capture data, *gazeStream* for data eye gaze data, etc. The hierarchy can be extended to support new data modalities by defining new classes that descend from any existing class.

Data stream objects are organized in a hierarchy with base class *dataStream;* this class defines some methods that operate on generic time series data, including *resample*, *filter*, and *plot*. Deriving from the *dataStream* base object allows straightforward definition of modality-specific data stream objects including objects that handle EEG, motion capture, or eye tracking data, among others. In the specific case of EEG data, the class *eeg* is defined to also derive from the class *headModel*, allowing integration of spatial anatomical information with functional information contained in the EEG time series (see Figure 2).

## Collection and synchronization of multiple data streams by LSL

MoBILAB itself is not meant for use during data collection, but for off-line data exploration. When reading multi-stream data from.*xdf* files, MoBILAB makes use of the time synchronization provided by the LSL (Lab Streaming Layer, referenced above) acquisition system. LSL implements time synchronization of concurrently recorded data streams by associating every collected sample with a timestamp provided by the high-resolution clock of the local computer on which the LSL data recorder application is running. When multiple data streams are recorded, LSL also estimates and stores the relative clock offsets among them. In general, multiple data streams may be collected concurrently via different computers located on a local area network, any one (or more) of which may serve to save the synchronized data to disk as an.*xdf* file that can then be imported into MoBILAB for review and processing. As it reads the file (using LSL function *load_xdf.m*), MoBILAB corrects the data stream timestamps by, first, estimating an (assumed) linear fit through the (possibly noisy) clock offsets and then correcting the time stamps for implied bias and drifts.

In practice, LSL can achieve millisecond precision or better synchronization, though only when the (non-zero) delay *within* each data acquisition device, from data sample capture to network sample transmission, is measured and made available to LSL in a device delay information file. Device delay information is most often best learned by direct measurement of each device. For example, the sample delay we have measured within our laboratory Biosemi Active 2 EEG system is 8 ms. When device delay information is available, LSL also corrects the.*xdf* file data stream timestamps for the measured delays, thereby achieving maximum stream synchronization accuracy while relieving.*xdf* data read functions of the need to locate and make use of this information.

## Memory management

In contrast to the common practice of representing data streams as matrix variables in the MATLAB workspace with computer random access memory (RAM), MoBILAB stores its working data in MATLAB memory-mapped data objects. Such memory-mapped objects are used, for example, by SPM (Friston et al., 1994) to represent MRI data. These objects organize the data in disk files optimized for fast random access, making it possible for MoBILAB to process data streams of virtually any size without requiring the host computer to have an unlimited amount of RAM, with relatively little compromise of compute performance (particularly as solid state and other disk access latencies continue to decrease). Each stream object is created from two files: (1) a.hdr header file that contains stream object metadata in MATLAB format, and (2) a.*bin* binary file that contains the data stream samples. Based on information in the header file, portions of the binary file are mapped into main memory only as and when needed. These operations are transparent to both MATLAB users and programmers. Data samples can be accessed using standard MATLAB and EEGLAB data matrix syntax through the *dataStream* object property *data*. Thus, MATLAB syntax for accessing data in EEGLAB and MoBILAB datasets appears similar:

$$\begin{array}{l} >>this\_data=EEG.data;\;\%\;copy\;EEGLAB\text{ ``}EEG^{\prime \prime }\;structure\\ \;\%\;dataintoa\;MATLAB\;matrix\\ >>this\_data=eegObj.data;\;\%\;copy\;MoBILAB\text{ ``}eegObj^{\prime \prime }\;object\\ \;\%\;data\;into\;a\;MATLAB\;matrix \end{array}$$

In the example above note that—contrary to the EEGLAB convention—MoBILAB represents data using samples as the first dimension and channels as the second (the transpose of *EEG.data*). The reason for this design choice is that MATLAB represents matrices internally as column-wise vectorized arrays. This is because operations performed channel-by-channel (for example, spectral filtering, correlations, etc.), run faster if channel samples are next to each other in RAM or on disk, as modern systems cache values before processing them.

Currently MoBILAB includes object classes to read/write, analyze, and visualize EEG, motion capture, eye tracking, and force plate data as well as recorded audio and/or video scene data. These classes include methods to apply spectral filters, compute temporal derivatives, and perform wavelet time-frequency decomposition, independent component analysis (ICA), or principal component analysis (PCA), plus some methods for electrical forward head modeling and inverse EEG source modeling not discussed here.

## The MoBILAB multi-stream browser

In MoBI experiments, the ability to review, interactively, the EEG data together with any other synchronously acquired data streams is critical for selecting, performing, and evaluating data measured during a MoBI experiment and for determining an appropriate data analysis path. The MoBILAB Multi-Stream Browser allows visual inspection, annotation, and sanity checking of recorded multistream data, and can also provide useful insights about suspected or previously unknown relationships between behavioral, environmental, and EEG data features. Each *dataStream* object has a built-in plotting function (a *plot* method) that displays the data stream in a natural manner. For some streams, more than one plotting method is supported to provide the benefits of different types of visualization. For instance, EEG may be displayed as a scrolling multi-channel time series or as an animated topographic scalp map, body motion capture data as movements of one or more 3-D stick figures, fixations in eye tracking data as time series or 2-D heat maps, etc. Extending MoBILAB plotting capabilities to a new type of *dataStream* is simple because core functions including data scrolling, interactive cursor actions, and other controls have been implemented in the base class *browserHandle* from which a programmer user can easily derive a new browser class by replacing and/or adding new elements that best suit the new data type.

**Figure 3:**
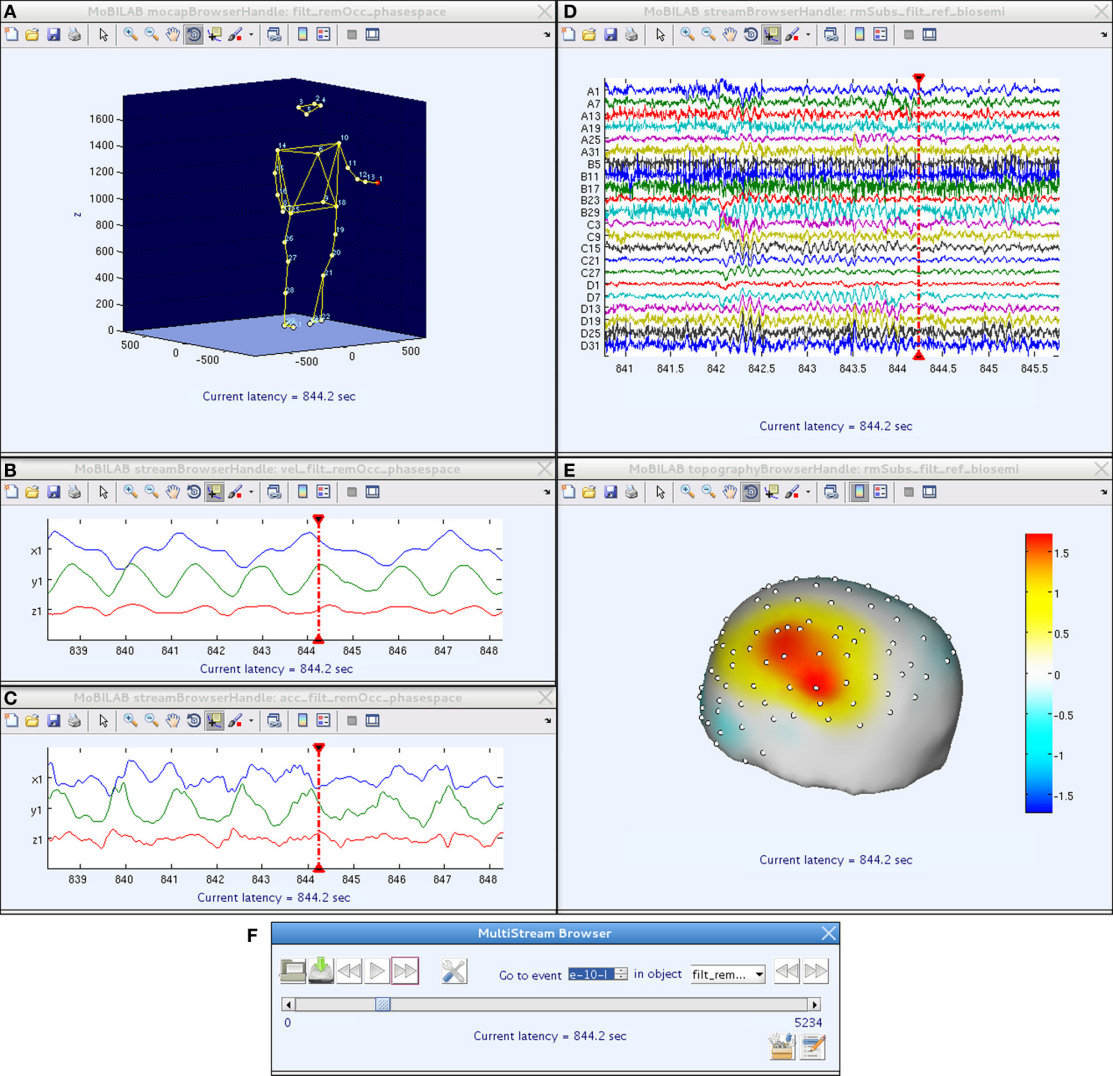
**A MoBILAB Multi-Stream Browser session comprising five (top) MATLAB figure windows that give user-selected, temporally coordinated views of raw and/or derived data streams within a multi-stream MoBI dataset, plus (bottom) a browser control window. (A)** Full body motion capture of participant's rhythmic hand and arm movements, animated on a 3-D human stick figure whose body position depicts subject position at the current moment (CM) in the data, marked in the scrolling time series data windows by CM indicators (vertical red lines). **(B,C)** Derived (x, y, z) velocity and acceleration time series for the (red) left hand marker in **(A)**, shown in a 10-s window containing the CM. **(D)** EEG time series for a 32-channel EEG channel subset, shown in a 5-s window containing the CM. **(E)** The EEG scalp topography at the CM, visualized by interpolating the channel EEG data on a template head model. **(F)** The Multi-Stream Browser control window. Movement of the CM in all browser windows at once can be controlled either by manipulating the play buttons or scroll bar in this window or by moving the red vertical CM indicator in any scrolling data window. Pressing play will animate the stick figure and scalp map displays to match the advancing CM. Data from (Leslie et al., in press).

Though each *dataStream* object can be visualized separately, either from the MoBILAB GUI or from the command line, when plotted through the Multi-Stream Browser multiple browser windows can be controlled from a single GUI (Figure 3). This control window performs user-directed scrolling through multimodal data (and/or through data streams derived from such data) either for multimodal data review and/or to mark experimental events of interest for further analysis.

Although when the Multi-Stream Browser is not meant for on-line data viewing, it is similar in spirit to Video-EEG monitoring systems used in epilepsy or sleep studies. However, unlike the MoBI data, in these systems the data are mostly recorded for clinical purposes and not always analyzed in great detail. As a complement, MoBILAB could be used in those cases where a more sophisticated analysis is required, as it provides the tools needed for multimodal data processing and exploration.

## Data provenance

**Figure 4:**
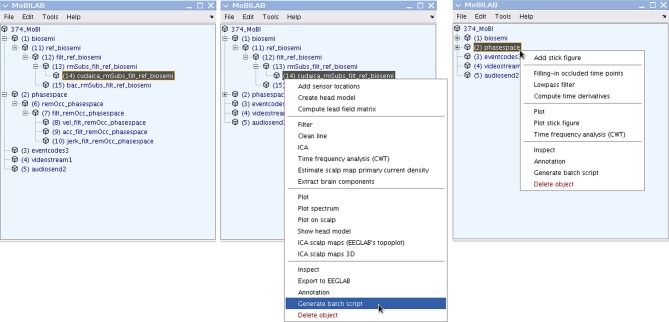
**The MoBILAB GUI and processing pipelines.** The left panel shows the tree of parent-children relationships among data objects in a loaded multi-stream MoBI dataset. The integers enclosed in parentheses to the left of each object name give the index in the cell array *mobilab.allStream.item*. The two branches shown unfolded (by clicking on the data object names, here *biosemi* and *phasespace*) represent two already selected data processing pipelines (cf. Text Boxes 1, 2): (1) for Biosemi (Amsterdam) system EEG data: raw data ⇒ re-referencing ⇒ high-pass filtering ⇒ artifact rejection ⇒ ICA decomposition; (2) for PhaseSpace (Inc.) system motion capture data: raw data ⇒ correction of occlusion artifacts ⇒ low-pass filtering ⇒ computation of time derivatives. By following any branch backwards (upwards), the user can generate MATLAB scripts that make it easy to run the same series of operations on other Biosemi and PhaseSpace datasets. The center panel shows the contextually defined menu for the EEG *dataStream* object. The menu item shaded in blue backtracks the history of every object in the selected branch, creating a script ready to run (see Text Box 1). The right panel shows the contextually defined menu for the motion capture data object (see Text Box 2). Note that the two stream objects (EEG and motion capture) have different processing menus that present their individually defined processing methods.

Figure 4 shows two sample MoBILAB GUI pipelines: (1) to process EEG data: raw data ⇒ re-referencing ⇒ high-pass filtering ⇒ artifact rejection ⇒ ICA decomposition; (2) to process motion capture data: raw data ⇒ occlusion artifact cleaning ⇒ low pass filtering ⇒ computation of time derivatives. Central to MoBILAB's design is its built-in data provenance, that gives users the ability to track and recall all the transformations applied to the data in a processing pipeline. Every stream object has a *history* property that is initialized at the moment of its creation with the command and the parameters that were used to generate it. This mechanism allows representing pipelines in which child datasets are processed versions of a parent set. Graphically, parent-child links can be visualized as a tree. To make this tree as functional and interactively accessible as possible, MoBILAB embeds the Java component *JTree* in the main MoBILAB GUI. This *JTree* component allows the creation of contextual menus for each data object in the tree. By climbing back up any branch of the tree (using menu item “*Generate batch script*”), the user can generate scripts that ease the task of running the same sequence of operations on other datasets.

Text BOX 1 **Automatically generated script to run an EEG data processing pipeline (from reading an XDF-formatted MoBI dataset to performing ICA decomposition) as generated by the MoBILAB EEG menu item “*Generate batch script*” in Figure 4 (center).**

Each command that modifies the data outputs a new object that handles the processed data; this object is also inserted into the MoBILAB object tree. Therefore, in the example below, *eegObj* is used as a temporary reference to the latest processed EEG dataset.

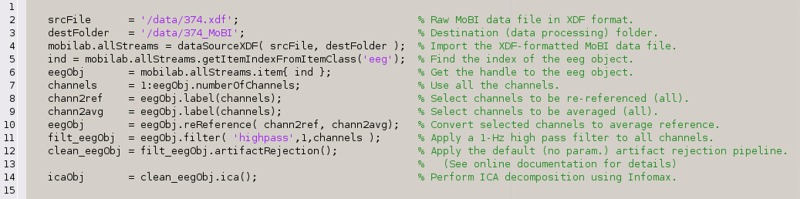

Text BOX 2 **A script implementing a MoBILAB pipeline for processing motion capture (“mocap”) data (from stream separation to computing time derivatives).**

This script was also generated using the menu item “*Generate batch script*” in Figure 4 (right).

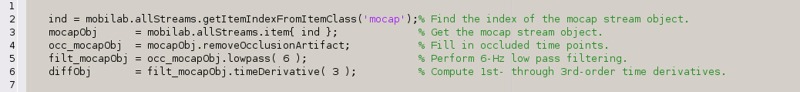

Other toolboxes including Brainstorm (Tadel et al., 2011) have successfully exploited this approach, making the user's interaction with the application simpler and more natural feeling. In multimodal data analysis, each data stream may have a different set of processing or pre-processing methods. For ease of use, therefore, MoBILAB provides a flexible menu interface that offers (only) selections relevant to each type of data stream present in the data.

The MoBILAB tree is meant to ease the analysis and exploration of different data types by exposing modality-specific standard options for visualization and analysis. Joint analysis of multi-rate MoBI data is still in an early stage, however. Though it is not yet possible to create converging multi-stream data processing pipelines from the GUI, it is possible for example to compute desired measures for more than one data stream and to then estimate their joint subject-level statistics through custom MoBILAB scripts, as demonstrated in (Leslie et al., in press).

## MoBILAB extensions

Developers can use the MoBILAB infrastructure (stream objects and its signal processing and visualization methods) as building blocks for rapid development of new MoBILAB extensions (formerly “plug-ins”). The example below shows a simple function that reconstructs the EEG channel data as the sum of only those of its independent components deemed to represent brain activity. One way of identifying “brain components” could be for instance to estimate the equivalent current dipole model of each independent component scalp map and then to select those components for which the residual variance of the scalp map not accounted for by the equivalent dipole model is less than some threshold.

The example below (in Text Box 4) shows how to create a class for a new type of *dataStream* object by re-using existing classes. The new class, named *icaEEG,* is intended to be a placeholder for the results of ICA decomposition applied to EEG data. It inherits all the properties and methods of the class *eeg* and adds properties to store the ICA field information from the EEGLAB *EEG* dataset structure. The first method defined is the so-called constructor; this function is called automatically by MATLAB at the moment of object creation. The constructor function is mandatory and has the same name as the class itself. The second method is described in Text Box 3. Integrating new functions and classes into the MoBILAB class hierarchy allows users to access the new methods directly from the contextual menu associated to each class. The third method uses the EEGLAB function *pop_topoplot* to display IC scalp maps. The fourth method shows how to redefine methods already defined in a base class. In this case, the method *EEGstructure* is extended to add ICA fields to the EEGLAB *EEG* structure.

Text BOX 3 **A MATLAB function to back-project to the scalp channels only those independent components of EEG data estimated to represent brain activity.**

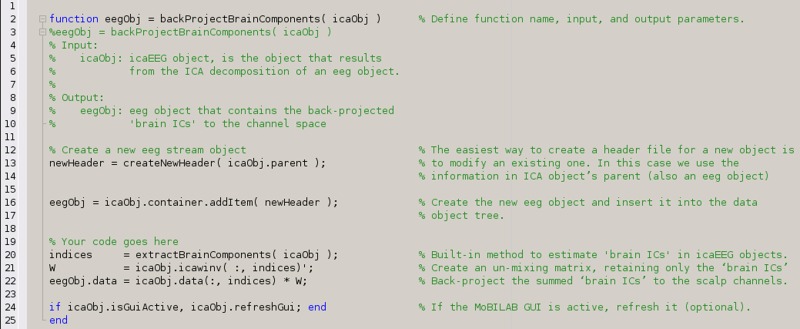

## EEG data processing

Although, as Text Box 4 illustrates, MoBILAB can export an EEG *dataStream* object from a multi-stream data file to EEGLAB as an *EEG* structure, it can also be used to pre-process and export an EEG dataset after performing ICA decomposition. MoBILAB also contains some EEG processing methods (under development) not yet available in EEGLAB itself.

Text BOX 4 **A definition of the new *dataStream* class *icaEEG* in MoBILAB.**

Observe that MATLAB requires the class to be defined in a m-file whose name matches the name of the class.

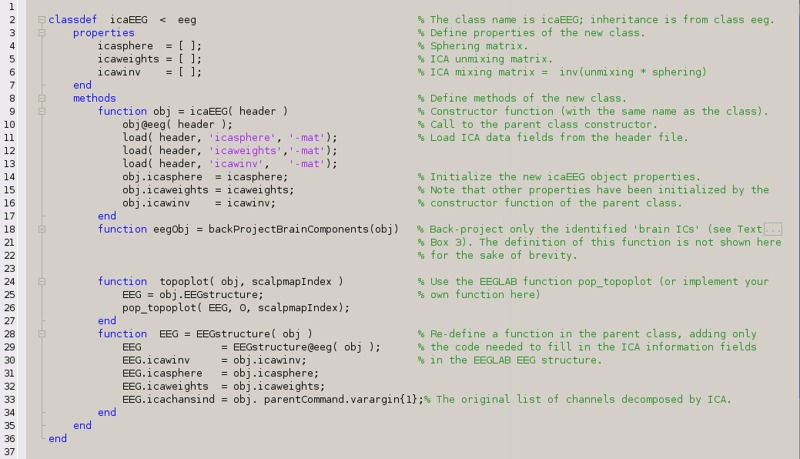

## Future directions

Here we have described MoBILAB, a software environment running on MATLAB for analysis and visualization of multimodal MoBI paradigm experiment data. MoBI analysis, and so also MoBILAB methods, are yet at an early stage of development. We therefore have limited our description to its general infrastructure, its Multi-Stream Browser, its motion capture data pre-processing facility, and its EEGLAB related features. At present, MoBILAB is a toolbox intended to provide researchers with basic tools for exploring their multimodal data. As the field of MoBI data analysis evolves, new methods will be added, specifically those for separately and jointly modeling brain and body dynamics using models incorporating more of the richness of the multimodal (brain/body/environment) MoBI data concept. Modeling brain dynamics while also taking into account body dynamics and interactions with environmental events (and other agents) should provide a better basis for understanding how the human brain supports our behavior and experience in the ever-changing context of daily life, thereby also gaining a deeper understanding of “how the brain works.”

A possible way to model brain/body/environment dynamics might be to extend the methodology of Dynamic Causal Modeling (Kiebel et al., 2009) first to the mechanics of the human body and then to its interface with the central nervous system (from spinal cord to the brain). Other approaches to MoBI data analysis might follow more data-driven approaches including those used in the field of BCI design (Makeig et al., 2012), whereby informative body and/or eye movement-defined events or features, extracted using body and eye movement models, might help classify and segregate EEG trials by cognitive state, response, or intention, thereby opening the possibility of adding powerful informative multimodal feature analysis to the repertoire of EEG/MoBI data analysis (as well as to BCI modeling). In this regard, we hope to strengthen ties between the MoBILAB, BCILAB (Kothe and Makeig, 2013), and SIFT (Delorme et al., 2011) toolboxes with a goal of better modeling EEG brain dynamics from multimodal data.

As a work in progress, new MoBILAB methods, bug fixes, and scripting examples will be added to existing documentation at sccn.ucsd.edu/wiki/MoBILAB. In the spirit of collaboration and openness that has characterized the development of EEGLAB and other open source scientific software projects, MoBILAB is freely available under open source BSD license. Explicit instructions for downloading and/or cloning the repository are given on the wiki. The authors would be pleased to collaborate with other interested researchers to extend the capabilities of MoBILAB to serve the evolving needs of MoBI brain research.

### Conflict of interest statement

The authors declare that the research was conducted in the absence of any commercial or financial relationships that could be construed as a potential conflict of interest.

## References

[B1] Delorme A. Makeig S. (2004). EEGLAB: an open source toolbox for analysis of single-trial EEG dynamics including independent component analysis. J. Neurosci. Methods 134, 9–21 10.1016/j.jneumeth.2003.10.009 15102499

[B2] Delorme A. Mullen T. Kothe C. Akalin Acar Z. Bigdely-Shamlo N. Vankov A. (2011). EEGLAB, SIFT, NFT, BCILAB, and ERICA: new tools for advanced EEG processing. Comput. Intell. Neurosci. 2011:130714 10.1155/2011/130714 21687590 PMC3114412

[B3] Friston K. J. Holmes A. P. Worsley K. J. Poline J.-P. Frith C. D. Frackowiak R. S. J. (1994). Statistical parametric maps in functional imaging: a general linear approach. Hum. Brain Mapp. 2, 189–210 10.1002/hbm.460020402

[B4] Gramann K. Gwin J. T. Ferris D. P. Oie K. Jung T.-P. Lin C.-T. (2011). Cognition in action: imaging brain/body dynamics in mobile humans. Rev. Neurosci. 22, 593–608 10.1515/RNS.2011.047 22070621

[B5] Gramfort A. Luessi M. Larson E. Engemann D. A. Strohmeier D. Brodbeck C. (2014). MNE software for processing MEG and EEG data. Neuroimage 86, 446–460 10.1016/j.neuroimage.2013.10.027 24161808 PMC3930851

[B6] Kiebel S. J. Garrido M. I. Moran R. Chen C.-C. Friston K. J. (2009). Dynamic causal modeling for EEG and MEG. Hum. Brain Mapp. 30, 1866–1876 10.1002/hbm.20775 19360734 PMC6870752

[B7] Kothe C. A. Makeig S. (2013). BCILAB: a platform for brain-computer interface development. J. Neural Eng. 10:056014 10.1088/1741-2560/10/5/056014 23985960

[B8] Leslie G. Ojeda A. Makeig S. (in press). Measuring musical engagement using expressive movement and EEG brain dynamics. Psychomusicology.

[B9] Makeig S. (1993). Auditory event-related dynamics of the EEG spectrum and effects of exposure to tones. Electroencephalogr. Clin. Neurophysiol. 86, 283–293 10.1016/0013-4694(93)90110-H 7682932

[B10] Makeig S. Bell A. J. Jung T.-P. Sejnowski T. J. (1996). “Independent component analysis of electroencephalographic data,” in Advances in Neural Information Processing Systems, eds Touretzky D. Mozer M. Hasselmo M. (Cambridge: MIT Press), 145–151

[B11] Makeig S. Debener S. Onton J. Delorme A. (2004). Mining event-related brain dynamics. Trends Cogn. Sci. 8, 204–210 10.1016/j.tics.2004.03.008 15120678

[B12] Makeig S. Gramann K. Jung T.-P. Sejnowski T. J. Poizner H. (2009). Linking brain, mind and behavior. Int. J. Psychophysiol. 73, 95–100 10.1016/j.ijpsycho.2008.11.008 19414039 PMC2796545

[B13] Makeig S. Kothe C. Mullen T. Bigdely-Shamlo N. Kreutz-Delgado K. (2012). Evolving signal processing for brain–computer interfaces. Proc. IEEE 100, 1567–1584 10.1109/JPROC.2012.218500923706528

[B14] Makeig S. Westerfield M. Jung T. P. Enghoff S. Townsend J. Courchesne E. (2002). Dynamic brain sources of visual evoked responses. Science 295, 690–694 10.1126/science.1066168 11809976

[B15] Oostenveld R. Fries P. Maris E. Schoffelen J.-M. (2011). FieldTrip: open source software for advanced analysis of MEG, EEG, and invasive electrophysiological data. Comput. Intell. Neurosci. 2011:156869 10.1155/2011/156869 21253357 PMC3021840

[B16] Schalk G. McFarland D. J. Hinterberger T. Birbaumer N. Wolpaw J. R. (2004). BCI2000: a general-purpose brain-computer interface (BCI) system. IEEE Trans. Biomed. Eng. 51, 1034–1043 10.1109/TBME.2004.827072 15188875

[B17] Schneider M. Wolpaw J. R. (2012). Brain-Computer Interfaces: Principles and Practice, eds Wolpaw J. R. Wolpaw E. W. (Oxford University Press) Available online at: http://www.amazon.com/Brain-Computer-Interfaces-Principles-Jonathan-Wolpaw/dp/0195388852

[B18] Sipp A. R. Gwin J. T. Makeig S. Ferris D. P. (2013). Loss of balance during balance beam walking elicits a multi-focal theta band electrocortical response. J. Neurophysiol. 110, 2050–2060 10.1152/jn.00744.2012 23926037 PMC3841925

[B19] Tadel F. Baillet S. Mosher J. C. Pantazis D. Leahy R. M. (2011). Brainstorm: a user-friendly application for MEG/EEG analysis. Comput. Intell. Neurosci. 2011:879716 10.1155/2011/879716 21584256 PMC3090754

[B20] Tallon-Baudry C. Bertrand O. Delpuech C. Pernier J. (1996). Stimulus specificity of phase-locked and non-phase-locked 40 Hz visual responses in human. J. Neurosci. 16, 4240–4249 8753885 10.1523/JNEUROSCI.16-13-04240.1996 PMC6579008

[B21] Verleger R. (1991). The instruction to refrain from blinking affects auditory P3 and N1 amplitudes. Electroencephalogr. Clin. Neurophysiol. 78, 240–251 10.1016/0013-4694(91)90039-7 1707797

[B22] Wang Y. Jung T.-P. (2013). “Improving brain–computer interfaces using independent component analysis,” in Towards Practical Brain-Computer Interfaces, eds Allison B. Z. Dunne S. Leeb R. Millán J. D. R. Nijholt A. (Berlin; Heidelberg: Springer), 67–83

